# Factors Associated with Hypertension Among Saudi Adults Attending Primary Healthcare Clinics: A Cross-Sectional Study

**DOI:** 10.3390/healthcare14213782

**Published:** 2026-11-05

**Authors:** Jumanah Muammar, Faisal Alomani, Ammar Abbas, Najd Aldakkan, Layan Aldoukhi, Abdulaziz Alsolimi, Kadi A. Alenzi, Sanaa S. Alanazi, Fairuz A. Algori, Haytham A. Sheerah

**Affiliations:** 1 Preventive Medicine Division, Prince Sultan Military Medical City, Riyadh 12233, Saudi Arabia; 2 Department of Family and Community Medicine, Riyadh Third Cluster, Riyadh 12233, Saudi Arabia; 3 College of Medicine, King Saud University, Riyadh 12233, Saudi Arabia; 4 Department of Internal Medicine, Prince Sultan Military Medical City, Riyadh 12233, Saudi Arabia; 5 Assistant Hospital Service Deputyship, Ministry of Health, Riyadh 12233, Saudi Arabia; 6 Nursing Department, Ministry of Health, Riyadh 12233, Saudi Arabia; 7 Population Health Deputyship, Ministry of Health, Riyadh 12233, Saudi Arabia; 8 International Collaboration Department, Chief Executive Office, Public Health Authority, Riyadh 12233, Saudi Arabia; 9 Preventive Medicine & Public Health, Faculty of Medicine, King Abdul-Aziz University, Jeddah 21589, Saudi Arabia

**Keywords:** hypertension, risk factors, body mass index, cardiovascular diseases, primary health care, Saudi Arabia

## Abstract

Background: Hypertension is a major public health challenge in Saudi Arabia. Previous studies have identified several sociodemographic, lifestyle, dietary, and clinical factors associated with hypertension, but findings have been inconsistent. Moreover, few studies have examined these factors simultaneously in primary healthcare settings. Therefore, this study examined their associations with hypertension among Saudi adults attending primary healthcare clinics. Methods: This cross-sectional study included 525 Saudi adults attending primary healthcare clinics at Prince Sultan Military Medical City, Riyadh, Saudi Arabia, in May 2026. Data were collected using a self-administered questionnaire, and hypertension status was verified using electronic medical records. Logistic regression was applied to identify factors associated with hypertension, and odds ratios (ORs) with 95% confidence intervals (CIs) were estimated. Results: Of the 525 participants, 146 (27.8%) had hypertension. After age and sex adjustment, advancing age, lower educational attainment, current smoking, electronic cigarette use, daily sugar-sweetened beverage consumption, higher body mass index (BMI), diabetes mellitus, hyperlipidemia, and history of cardiovascular disease were associated with hypertension. However, after multivariable adjustment, only advancing age (OR = 1.09, 95% CI: 1.06–1.12), higher BMI (OR = 1.07, 95% CI: 1.02–1.12), and history of cardiovascular disease (OR = 2.01, 95% CI: 1.01–4.06) remained associated with hypertension. Conclusions: These findings support closer blood pressure monitoring among older adults, individuals with higher BMI, and those with a history of cardiovascular disease in primary healthcare settings. Weight management should also be considered an important component of hypertension prevention.

## 1. Introduction

Hypertension remains a public health challenge despite advances in its prevention and treatment [1,2]. It is a leading risk factor for cardiovascular disease and premature mortality worldwide. In 2019, an estimated 1.28 billion adults aged 30–79 years were living with hypertension, and elevated blood pressure was responsible for approximately 8.5 million deaths [3]. Persistent elevation of blood pressure substantially increases the risk of coronary artery disease, stroke, heart failure, chronic kidney disease, and premature death [2]. Beyond its health consequences, hypertension imposes a substantial economic burden, with direct healthcare costs exceeding USD 1400 per patient, in addition to large productivity losses and other indirect costs [4].

Saudi Arabia has experienced a substantial increase in the burden of non-communicable diseases, including hypertension, over recent decades [5]. National and regional studies have consistently shown that hypertension affects a substantial proportion of Saudi adults, although reported prevalence estimates vary according to study population, geographical region, and diagnostic criteria, ranging from approximately 5% to over 30%. National surveys have also demonstrated suboptimal levels of awareness, treatment, and blood pressure control [6,7,8,9,10,11,12,13,14,15,16,17,18,19,20,21,22,23,24,25,26,27,28,29].

Recent systematic reviews have synthesized the available evidence on factors associated with hypertension among Saudi adults [30,31]. Advancing age and obesity have consistently emerged as the strongest determinants across national and regional studies. Other frequently reported factors include male sex, lower educational attainment, diabetes mellitus, hyperlipidemia, cardiovascular disease, smoking, physical inactivity, and unhealthy dietary behaviors, particularly high salt intake and inadequate consumption of fruits and vegetables [30,31]. Nevertheless, the associations observed for several lifestyle, dietary, and socioeconomic factors have been inconsistent, with some studies reporting significant associations while others found no independent relationship after adjustment for potential confounders [30,31]. These inconsistencies may reflect differences in study populations, geographical settings, sampling strategies, variable definitions, and analytical approaches.

Although numerous Saudi studies have investigated hypertension, many have focused on selected demographic, lifestyle, or clinical factors rather than examining them simultaneously. Furthermore, much of the available evidence comes from community-based or general population studies, while evidence from primary healthcare settings remains limited. Patients attending primary healthcare clinics may differ from the general population in their demographic and clinical characteristics. Therefore, the present study aimed to examine the sociodemographic, lifestyle, dietary, and clinical factors associated with hypertension among Saudi adults attending primary healthcare clinics in Saudi Arabia.

## 2. Methods

### 2.1. Study Design, Setting, and Study Population

This cross-sectional study was conducted among adults attending the outpatient primary healthcare clinics of Prince Sultan Military Medical City, Riyadh, Saudi Arabia. Participants were recruited from the waiting areas of the primary healthcare clinics in May 2026. Individuals were eligible to participate if they were Saudi nationals, aged ≥ 18 years, and were able to provide informed consent.

### 2.2. Sample Size Calculation

The required sample size was estimated using the single population proportion formula for cross-sectional studies (n = Z^2^p(1 − *p*)/d^2^), where (n) is the required sample size, (Z) is the standard normal deviate corresponding to the desired confidence level (1.96 for 95% confidence), (*p*) is the anticipated prevalence of hypertension (25%), and (d) is the margin of error (5%). Assuming a hypertension prevalence of approximately 25% among Saudi adults based on a previous nationwide study [8], a confidence level of 95%, a margin of error of 5%, and a design effect of one, the minimum required sample size was calculated to be 288 participants. To account for incomplete questionnaires and improve the precision of the estimates, a larger sample was recruited. A total of 525 participants completed the study and were included in the final analysis.

### 2.3. Data Collection Tool

Data were collected using a structured, self-administered Arabic questionnaire that was developed after reviewing the relevant literature on hypertension and its associated risk factors. The questionnaire collected information on sociodemographic characteristics, including age, sex, marital status (single, married, divorced, or widowed), educational level (less than secondary, secondary, diploma, university, or postgraduate), employment status (employed, unemployed, student, or retired), and monthly household income (<5000, 5000–9999, 10,000–14,999, or ≥15,000 Saudi Riyals). Lifestyle characteristics included cigarette smoking status (never, former, or current smoker), electronic cigarette use (yes or no), physical activity, sleep duration, and body weight and height. Body mass index (BMI) was calculated as weight in kilograms divided by the square of height in meters (kg/m^2^). Weekly physical activity was categorized as no physical activity, <60 min/week, 60–149 min/week, or ≥150 min/week [32]. Average daily sleep duration was categorized as <5 h, 5–6 h, 7–8 h, or >8 h [33]. Dietary information included the usual frequency of fruits, vegetables, fish, sugar-sweetened beverages, and salty or pickled food consumption. Fruit and vegetable intake were categorized as none/rarely, once/day, twice/day, or three or more times/day. Fish consumption was categorized as none/rarely, once/week, or two or more times/week [34]. Sugar-sweetened beverage consumption was classified as none/rarely, 1–2 times/week, 3–6 times/week, or daily, whereas salty or pickled food consumption was categorized as none/rarely, sometimes, or always. Participants were also asked whether they had a physician diagnosis of diabetes mellitus, hyperlipidemia, or cardiovascular disease. Questionnaires were reviewed on-site by the research team for completeness. Participants were asked to complete any missing responses before leaving.

The primary outcome was hypertension. Hypertension status was initially determined based on participants’ self-report of a previous physician diagnosis. To improve the accuracy of outcome ascertainment, participants’ electronic medical records were reviewed. Participants were classified as having hypertension if they had a documented physician diagnosis of hypertension, a recorded systolic blood pressure of ≥140 mmHg and/or diastolic blood pressure of ≥90 mmHg measured on two separate clinical encounters [35], or documentation of current antihypertensive medication use in their medical records.

### 2.4. Statistical Analysis

Statistical analyses were performed using IBM SPSS Statistics version 23 (IBM Corp., Armonk, NY, USA). Continuous variables were summarized using means and standard deviations (SD), whereas categorical variables were presented as frequencies and percentages. Separate age- and sex-adjusted logistic regression models were fitted to examine the association between each sociodemographic, lifestyle, dietary, and clinical factor and hypertension. Variables associated with hypertension at *p*-values < 0.05 in previous models were entered into the multivariable logistic regression model together with age and sex. Odds ratios (ORs) and corresponding 95% confidence intervals (CIs) were reported, and a two-sided *p*-value < 0.05 was considered statistically significant.

## 3. Results

### 3.1. Participant Characteristics

A total of 525 Saudi adults were included in the study, of whom 146 (27.8%) had hypertension. All 146 participants with hypertension had at least two of the following findings: elevated blood pressure, self-reported history of hypertension, or self-reported use of antihypertensive medications.

The mean age of the participants was 44.46 ± 12.85 years, and 55.8% were women. The mean BMI was 28.92 ± 5.57 kg/m^2^. Most participants were married (76.4%), while 41.1% were employed and 40.8% were unemployed. Regarding lifestyle characteristics, 16.4% of participants were current smokers, 11.4% were former smokers, and 2.3% reported electronic cigarette use. More than half (53.5%) reported no physical activity, whereas only 25.7% achieved at least 150 min of physical activity per week. With respect to dietary habits, 18.7% consumed sugar-sweetened beverages daily, 14.9% always consumed salty or pickled foods, 55.8% reported no or rare fruit consumption, 24.2% reported no or rare vegetable consumption, and 66.1% reported no or rare fish consumption. Diabetes mellitus, hyperlipidemia, and a history of cardiovascular disease were reported by 37.9%, 51.2%, and 10.1% of participants, respectively (Table 1).

Hypertension prevalence increased with age, from 7.2% among participants aged 20–39 years to 20.3% among those aged 40–49 years, 47.6% among those aged 50–59 years, and 64.4% among those aged ≥ 60 years (Table S1). In addition, hypertension prevalence increased across BMI categories, from 11.1% among underweight participants to 17.6% among those with normal BMI, 25.9% among overweight participants, and 36.1% among obese participants (Table S2).

### 3.2. Sociodemographic Factors Associated with Hypertension (Age- and Sex-Adjusted)

Participants with hypertension were older than those without hypertension (54.29 ± 10.52 vs. 40.67 ± 11.61 years), and each one-year increase in age was associated with higher odds of hypertension (OR = 1.10, 95% CI: 1.08–1.13). Participants with a diploma or lower educational level had higher odds of hypertension than those with university or postgraduate education (OR = 2.28, 95% CI: 1.15–4.51). No significant associations were observed for sex, marital status, employment status, or monthly income (Table 2).

### 3.3. Lifestyle Factors Associated with Hypertension (Age- and Sex-Adjusted)

Current smokers had higher odds of hypertension than never smokers (OR = 2.05, 95% CI: 1.02–4.11). Electronic cigarette use was also associated with higher odds of hypertension (OR = 4.32, 95% CI: 1.15–16.27). No significant associations were observed between hypertension and physical activity level or sleep duration (Table 3).

### 3.4. Dietary Factors Associated with Hypertension (Age- and Sex-Adjusted)

Daily consumption of sugar-sweetened beverages was associated with higher odds of hypertension compared with participants who did not consume sugar-sweetened beverages (OR = 2.21, 95% CI: 1.17–4.17). Fruit consumption, vegetable consumption, fish consumption, and salty or pickled food consumption were not associated with hypertension (Table 4).

### 3.5. Clinical Factors Associated with Hypertension (Age- and Sex-Adjusted)

Higher BMI (OR = 1.07, 95% CI: 1.03–1.12), diabetes mellitus (OR = 1.75, 95% CI: 1.06–2.89), hyperlipidemia (OR = 1.79, 95% CI: 1.08–2.97), and a history of cardiovascular disease (OR = 2.30, 95% CI: 1.17–4.50) were significantly associated with hypertension (Table 5).

### 3.6. Multivariable Logistic Regression Analysis

In the multivariable model, only advancing age (OR = 1.09, 95% CI: 1.06–1.12), higher BMI (OR = 1.07, 95% CI: 1.02–1.12), and history of cardiovascular disease (OR = 2.01, 95% CI: 1.01–4.06) remained significantly associated with hypertension. Educational level, smoking status, electronic cigarette use, sugar-sweetened beverage consumption, diabetes mellitus, and hyperlipidemia were not significantly associated with hypertension (Table 6).

## 4. Discussion

In this cross-sectional study of Saudi adults attending primary healthcare clinics, approximately one in four participants had hypertension. Although several sociodemographic, lifestyle, dietary, and clinical factors were associated with hypertension in age- and sex-adjusted analyses, only advancing age, higher BMI, and a history of cardiovascular disease remained associated with hypertension in the multivariable model.

Our estimated prevalence (27.8%) falls within the wide range previously reported in Saudi Arabia. National surveys have consistently demonstrated that hypertension affects a substantial proportion of Saudi adults, although prevalence estimates vary according to the study population, geographical region, age distribution, and diagnostic criteria. A national survey conducted in 2013 reported a prevalence of 15.2% [6], whereas an earlier nationwide survey based on the WHO STEPwise approach estimated that approximately one quarter of Saudi adults had hypertension and highlighted low levels of awareness, treatment, and blood pressure control [7,8]. More recent nationwide studies have reported prevalence estimates ranging from 6.5% to 12.7% [9,10,11,12]. Regional and community-based studies have also reported considerable variation, with prevalence estimates ranging from 4.9% in Al-Kharj [13] and 11% in Riyadh [14] to 22.6% in Jeddah [15], 25.5% in Hawtat Bani Tamim [16], and 34.1% in Taif [17]. Additional studies conducted in Jeddah, Madinah, Riyadh, the Eastern Province, Aseer, and Tabuk have further demonstrated substantial regional variation in hypertension prevalence [18,19,20,21,22,23,24,25,26,27,28,29].

The prevalence of hypertension in our study was also slightly lower than international estimates. According to the WHO 2025 hypertension profiles, the estimated prevalence among adults aged 30–79 years in 2024 was 34% worldwide, compared with 30% in Saudi Arabia, 32% in Qatar, 32% in Bahrain, 35% in the United Arab Emirates, 37% in Oman, and 39% in Kuwait [36]. However, these estimates are not directly comparable with our findings because of differences in age distribution, sampling methods, and study settings. Methodological differences may also explain variations in hypertension prevalence between studies. Studies relying on self-reported diagnoses, single-visit blood pressure measurements, or medical records may produce different estimates. In our study, 39.4% of participants were aged 20–39 years, and hypertension prevalence increased from 7.2% in this age group to 64.4% among those aged ≥ 60 years. Therefore, the relatively lower overall prevalence may partly reflect the younger age distribution of our participants. Furthermore, our study was conducted at a single primary healthcare center and may not represent the general Saudi population.

Advancing age was strongly associated with hypertension in the present study. Each one-year increase in age was associated with approximately 9% higher odds of hypertension after multivariable adjustment. This finding is consistent with previous Saudi and international studies, which have identified advancing age as the most important non-modifiable determinant of hypertension across diverse populations, including national surveys, community-based studies, and primary healthcare settings [6,7,8,9,10,11,12,13,14,15,16,17,18,19,20,21,22,23,24,25,26,27,28,29,30,31,32,33,34,35,36,37,38,39]. Age-related arterial stiffening, endothelial dysfunction, increased vascular resistance, and reduced arterial compliance contribute to progressive elevations in systolic blood pressure, while prolonged exposure to behavioral and metabolic risk factors further accelerates hypertension development [40,41]. Given the rapidly ageing Saudi population, these findings emphasize the importance of regular blood pressure screening and early detection among older adults.

Higher BMI also remained independently associated with hypertension after adjustment for multiple covariates, with each 1 kg/m^2^ increase corresponding to a 7% increase in the odds of hypertension. Obesity has consistently emerged as one of the strongest determinants of hypertension in previous Saudi studies [6,8,13,14,15,16,17,18,19,20,30,31]. Several biological mechanisms may explain this relationship. Obesity promotes sympathetic nervous system activation, stimulates the renin–angiotensin–aldosterone system, increases renal sodium reabsorption, impairs endothelial function, and induces chronic low-grade inflammation, all of which contribute to sustained elevations in blood pressure [42,43]. From a public health perspective, this finding is particularly important because obesity is highly prevalent in Saudi Arabia. Recent nationwide studies estimated that approximately one in five Saudi adults were living with obesity, with prevalence remaining relatively stable between 21.4% and 22.2% during 2020–2023 [44,45]. Unlike age, obesity is a potentially modifiable risk factor. Strategies that promote healthy weight through dietary modification, regular physical activity, and pharmacological therapies can reduce the burden of hypertension and its cardiovascular complications [46,47,48].

Having a history of cardiovascular disease was the third factor that remained independently associated with hypertension. Individuals with previous cardiovascular disease had approximately twice the odds of hypertension compared with those without cardiovascular disease. Hypertension and cardiovascular disease frequently coexist because they share multiple cardiometabolic risk factors, while hypertension itself is a major contributor to the development and progression of cardiovascular disease. Long-standing hypertension contributes to atherosclerosis, left ventricular hypertrophy, heart failure, and ischemic heart disease, whereas individuals with established cardiovascular disease usually have multiple coexisting cardiometabolic risk factors that predispose them to persistent hypertension [49,50]. Similar associations have been reported in previous Saudi studies that demonstrated a clustering of hypertension with other cardiovascular risk factors [51,52]. These findings further support the recommendations of the National Heart Center and Saudi Heart Association advocating blood pressure control among individuals with established cardiovascular disease [53].

Several factors, including lower educational attainment, current cigarette smoking, electronic cigarette use, daily consumption of sugar-sweetened beverages, diabetes mellitus, and hyperlipidemia, were associated with hypertension after adjustment for age and sex but did not remain significant in the multivariable model. This does not necessarily indicate that these factors are unrelated to hypertension. Their associations may have been influenced by other factors included in the model, particularly age and BMI. In addition, the relatively small number of participants with hypertension may have limited the ability to detect some associations. Physical activity and dietary habits were assessed using self-reported questions, which may have introduced reporting errors and reduced the ability to identify associations. The absence of significant associations with some lifestyle factors should not be interpreted as evidence of healthier behaviors among Saudi adults. In fact, 53.5% of our participants reported no physical activity, 39.6% were obese, and 55.8% reported no or rare fruit consumption. These findings suggest that unhealthy lifestyle behaviors remain common in our study population despite their lack of independent associations with hypertension. They also highlight the complex interrelationships among behavioral, metabolic, and socioeconomic factors involved in hypertension development [54].

This study has several limitations that should be acknowledged. First, the cross-sectional design does not allow causal inference or the establishment of temporal relationships between the investigated factors and hypertension. Reverse causation also cannot be excluded, particularly for cardiovascular disease history and lifestyle behaviors that may have changed following a hypertension diagnosis. Second, the study was conducted at a single primary healthcare center in Riyadh, which may limit the generalizability of the findings to other healthcare settings, geographical regions, and the general Saudi population. Third, several variables, including smoking status, physical activity, dietary habits, body weight, height, and physician-diagnosed comorbidities, were self-reported and therefore subject to recall and reporting bias. Although hypertension status was verified using electronic medical records, potential misclassification of the self-reported variables may have influenced the observed associations. Fourth, dietary intake was assessed using a brief questionnaire that did not quantify portion sizes or total nutrient intake, which may have limited our ability to detect associations between dietary factors and hypertension. Fifth, information on specific cardiovascular diseases, such as coronary artery disease, heart failure, and atrial fibrillation, was not collected. Therefore, we could not examine the associations of individual cardiovascular conditions with hypertension. Sixth, information on participants’ place of residence (urban or rural) was not collected. Although the study was conducted at a primary healthcare center in an urban area, the residential background of individual participants could not be confirmed. Seventh, waist circumference and other measures of body composition were not collected. Therefore, we could not assess abdominal obesity or distinguish fat mass from fat-free mass using BMI alone. Eighth, cognitive function was not formally assessed. However, given that only 3.8% of participants were older than 65 years, age-related cognitive impairment was unlikely to have had a major impact on the study findings, although its potential influence cannot be excluded. Ninth, we used predefined cutoffs for physical activity, sleep duration, and dietary habits, which were not all based on established guidelines. Furthermore, physical activity intensity and dietary portion sizes were not assessed, limiting our ability to evaluate adherence to standard recommendations. These classifications may have introduced exposure misclassification and reduced our ability to detect associations with hypertension.

## 5. Conclusions

Hypertension was common among Saudi adults attending primary healthcare clinics, affecting more than one quarter of the study population. Advancing age, higher BMI, and a history of cardiovascular disease were independently associated with hypertension, highlighting the importance of targeting high-risk individuals for screening and preventive interventions. Strategies aimed at promoting healthy weight and comprehensive cardiovascular risk management may contribute substantially to reducing the burden of hypertension. Prospective multicenter studies are warranted to further elucidate the complex interplay between lifestyle, metabolic, and sociodemographic factors in the development of hypertension.

## Figures and Tables

**Table 1:** Characteristics of the study participants (N = 525).

Variable	Category	Overall, n (%)
Age, years	Mean ± SD (range)	44.46 ± 12.85 (20–70)
Systolic blood pressure, mmHg	Mean ± SD (range)	122.47 ± 16.58 (70–182)
Body mass index, kg/m^2^	Mean ± SD (range)	28.92 ± 5.57 (13.89–51.03)
Sex	Men	232 (44.2)
	Women	293 (55.8)
Marital status	Single	75 (14.3)
	Married	401 (76.4)
	Divorced	24 (4.6)
	Widowed	25 (4.8)
Educational level	Less than secondary	138 (26.3)
	Secondary	163 (31.0)
	Diploma	97 (18.5)
	University	116 (22.1)
	Postgraduate	11 (2.1)
Employment status	Employed	216 (41.1)
	Student	11 (2.1)
	Retired	84 (16.0)
	Unemployed	214 (40.8)
Monthly income, SAR	<5000	112 (21.3)
	5000–9999	200 (38.1)
	10,000–14,999	145 (27.6)
	≥15,000	68 (13.0)
Fruit consumption	None/Rarely	293 (55.8)
	Once/day	177 (33.7)
	Twice/day	40 (7.6)
	≥3 times/day	15 (2.9)
Vegetable consumption	None/Rarely	127 (24.2)
	Once/day	277 (52.8)
	Twice/day	94 (17.9)
	≥3 times/day	27 (5.1)
Fish consumption	None/Rarely	347 (66.1)
	Once/week	121 (23.0)
	≥2 times/week	57 (10.9)
Sugar-sweetened beverages	None/Rarely	240 (45.7)
	1–2/week	118 (22.5)
	3–6/week	69 (13.1)
	Daily	98 (18.7)
Salty or pickled food consumption	None/Rarely	425 (81.0)
	Sometimes	22 (4.2)
	Always	78 (14.9)
Smoking status	Never	379 (72.2)
	Former	60 (11.4)
	Current	86 (16.4)
Electronic cigarette use	No	513 (97.7)
	Yes	12 (2.3)
Physical activity	None	281 (53.5)
	<60 min/week	35 (6.7)
	60–149 min/week	74 (14.1)
	≥150 min/week	135 (25.7)
Sleep duration	<5 h/day	80 (15.2)
	5–6 h/day	203 (38.7)
	7–8 h/day	217 (41.3)
	>8 h/day	25 (4.8)
Hypertension	No	379 (72.2)
	Yes	146 (27.8)
Diabetes mellitus	No	326 (62.1)
	Yes	199 (37.9)
Hyperlipidemia	No	256 (48.8)
	Yes	269 (51.2)
History of cardiovascular disease	No	472 (89.9)
	Yes	53 (10.1)

**Table 2:** Sociodemographic factors associated with hypertension.

Variable	Category	No Hypertension (n = 379)	Hypertension (n = 146)	Adjusted OR (95% CI)
Age, years	Mean ± SD	40.67 ± 11.61	54.29 ± 10.52	1.10 (1.08–1.13)
Sex	Women	224 (59.1)	69 (47.3)	Reference
	Men	155 (40.9)	77 (52.7)	1.55 (1.00–2.41)
Marital status	Single	68 (17.9)	7 (4.8)	Reference
	Married	285 (75.2)	116 (79.5)	0.76 (0.29–1.99)
	Divorced	13 (3.4)	11 (7.5)	1.55 (0.44–5.41)
	Widowed	13 (3.4)	12 (8.2)	0.83 (0.22–3.13)
Educational level	University/postgraduate	115 (30.3)	12 (8.2)	Reference
	Diploma or lower	264 (69.7)	134 (91.8)	2.28 (1.15–4.51)
Employment status	Unemployed	160 (42.2)	54 (37.0)	Reference
	Employed	175 (46.2)	41 (28.1)	0.76 (0.39–1.48)
	Student	11 (2.9)	0 (0.0)	Not estimable
	Retired	33 (8.7)	51 (34.9)	1.14 (0.55–2.37)
Monthly income, SAR	<5000	67 (17.7)	45 (30.8)	Reference
	5000–9999	150 (39.6)	50 (34.2)	0.67 (0.37–1.20)
	10,000–14,999	114 (30.1)	31 (21.2)	0.61 (0.32–1.16)
	≥15,000	48 (12.7)	20 (13.7)	1.11 (0.52–2.37)

OR, odds ratio; CI, confidence interval. Odds ratios were adjusted for age and sex. Age was adjusted for sex only, and sex was adjusted for age only. The odds ratio for students was not estimable because no student had hypertension.

**Table 3:** Lifestyle factors associated with hypertension.

Variable	Category	No Hypertension (n = 379)	Hypertension (n = 146)	Adjusted OR (95% CI)
Smoking status	Never	286 (75.5)	93 (63.7)	Reference
	Former	35 (9.2)	25 (17.1)	1.85 (0.90–3.81)
	Current	58 (15.3)	28 (19.2)	2.05 (1.02–4.11)
Electronic cigarette use	No	372 (98.2)	141 (96.6)	Reference
	Yes	7 (1.8)	5 (3.4)	4.32 (1.15–16.27)
Physical activity	None	202 (53.3)	79 (54.1)	Reference
	<60 min/week	22 (5.8)	13 (8.9)	1.65 (0.69–3.95)
	60–149 min/week	61 (16.1)	13 (8.9)	0.48 (0.23–1.00)
	≥150 min/week	94 (24.8)	41 (28.1)	1.30 (0.75–2.24)
Sleep duration	>8 h/day	17 (4.5)	8 (5.5)	Reference
	7–8 h/day	156 (41.2)	61 (41.8)	0.59 (0.21–1.69)
	5–6 h/day	154 (40.6)	49 (33.6)	0.49 (0.17–1.40)
	<5 h/day	52 (13.7)	28 (19.2)	0.72 (0.23–2.21)

OR, odds ratio; CI, confidence interval. Odds ratios were adjusted for age and sex.

**Table 4:** Dietary factors associated with hypertension.

Variable	Category	No Hypertension (n = 379)	Hypertension (n = 146)	Adjusted OR (95% CI)
Fruit consumption	None	224 (59.1)	69 (47.3)	Reference
	Once/day	121 (31.9)	56 (38.4)	1.30 (0.80–2.09)
	Twice/day	24 (6.3)	16 (11.0)	1.89 (0.85–4.20)
	≥3 times/day	10 (2.6)	5 (3.4)	1.29 (0.37–4.44)
Vegetable consumption	None	94 (24.8)	33 (22.6)	Reference
	Once/day	195 (51.5)	82 (56.2)	1.40 (0.81–2.42)
	Twice/day	71 (18.7)	23 (15.8)	1.42 (0.69–2.91)
	≥3 times/day	19 (5.0)	8 (5.5)	1.69 (0.59–4.82)
Fish consumption	None	252 (66.5)	95 (65.1)	Reference
	Once/week	93 (24.5)	28 (19.2)	0.87 (0.50–1.51)
	≥2 times/week	34 (9.0)	23 (15.8)	1.86 (0.93–3.71)
Sugar-sweetened beverages	None	163 (43.0)	77 (52.7)	Reference
	1–2/week	93 (24.5)	25 (17.1)	0.82 (0.46–1.49)
	3–6/week	56 (14.8)	13 (8.9)	1.09 (0.49–2.41)
	Daily	67 (17.7)	31 (21.2)	2.21 (1.17–4.17)
Salty or pickled foods	Rarely	300 (79.2)	125 (85.6)	Reference
	Sometimes	16 (4.2)	6 (4.1)	1.08 (0.37–3.19)
	Always	63 (16.6)	15 (10.3)	0.79 (0.40–1.55)

OR, odds ratio; CI, confidence interval. Odds ratios were adjusted for age and sex.

**Table 5:** Clinical factors associated with hypertension.

Variable	Category	No Hypertension (n = 379)	Hypertension (n = 146)	Adjusted OR (95% CI)
Body mass index, kg/m^2^	Mean ± SD	28.33 ± 5.64	30.43 ± 5.09	1.07 (1.03–1.12)
Body mass index *	Normal	106 (28.0)	22 (15.1)	Reference
	Overweight	140 (36.9)	49 (33.6)	1.57 (0.81, 3.05)
	Obese	133 (35.1)	75 (51.3)	2.59 (1.36, 4.94)
Diabetes mellitus	No	277 (73.1)	49 (33.6)	Reference
	Yes	102 (26.9)	97 (66.4)	1.75 (1.06–2.89)
Hyperlipidemia	No	222 (58.6)	34 (23.3)	Reference
	Yes	157 (41.4)	112 (76.7)	1.79 (1.08–2.97)
History of cardiovascular disease	No	356 (93.9)	116 (79.5)	Reference
	Yes	23 (6.1)	30 (20.5)	2.30 (1.17–4.50)

OR, odds ratio; CI, confidence interval. Odds ratios were adjusted for age and sex. * Body mass index categories were defined as underweight (<18.5 kg/m^2^), normal weight (18.5–24.9 kg/m^2^), overweight (25.0–29.9 kg/m^2^), and obesity (≥30.0 kg/m^2^); however, underweight and normal weight were merged into one group (normal weight) because of the limited number of participants in the underweight group.

**Table 6:** Multivariable logistic regression analysis of factors associated with hypertension.

Variable	Category	Adjusted OR (95% CI)
Age (years)	Continuous	1.09 (1.06–1.12)
Sex	Men vs. women	1.23 (0.71–2.15)
Educational level	Diploma or lower vs. university/postgraduate	1.55 (0.74–3.21)
Smoking status	Former vs. never	1.52 (0.71–3.24)
	Current vs. never	1.25 (0.58–2.69)
Electronic cigarette use	Yes vs. no	3.78 (0.94–15.13)
Sugar-sweetened beverage consumption	1–2/week vs. never	0.71 (0.38–1.33)
	3–6/week vs. never	1.00 (0.43–2.31)
	Daily vs. never	1.83 (0.93–3.58)
Body mass index (kg/m^2^)	Continuous	1.07 (1.02–1.12)
Body mass index *	Overweight vs. normal	1.69 (0.83, 3.44)
	Obese vs. normal	2.78 (1.38, 5.60)
Diabetes mellitus	Yes vs. no	1.38 (0.80–2.38)
Hyperlipidemia	Yes vs. no	1.45 (0.84–2.52)
History of cardiovascular disease	Yes vs. no	2.01 (1.01–4.06)

OR, odds ratio; CI, confidence interval. Odds ratios were adjusted for age, sex, educational level, smoking status, electronic cigarette use, sugar-sweetened beverage consumption, body mass index, diabetes mellitus, hyperlipidemia, and history of cardiovascular disease. * Body mass index categories were defined as underweight (<18.5 kg/m^2^), normal weight (18.5–24.9 kg/m^2^), overweight (25.0–29.9 kg/m^2^), and obesity (≥30.0 kg/m^2^); however, underweight and normal weight were merged into one group (normal weight) because of the limited number of participants in the underweight group.

## Data Availability

The data presented in this study are available on request from the corresponding author due to ethical and administrative reasons.

## Supplementary Materials

The following supporting information can be downloaded at: https://www.mdpi.com/article/10.3390/healthcare14213782/s1. Table S1. Prevalence of hypertension by age group (N = 525). Table S2. Prevalence of hypertension by BMI category (N = 525).
