# Supplementary material for: Factors Associated with Hypertension Among Saudi Adults Attending Primary Healthcare Clinics: A Cross-Sectional Study
Source: Healthcare (Basel). 2026 Nov 5;14(21):3782. doi: 10.3390/healthcare14213782 (PMC13763097; doi:10.3390/healthcare14213782)
Supplement: Supplementary file 1 [file healthcare-14-03782-s001.zip › healthcare-4592495-supplementary.pdf]

**Table S1. Prevalence of hypertension by age group (N = 525).**

| Age group (years) | Total, n | Hypertension, n (%) |
|---|---|---|
| **20–39** | 207 | 15 (7.2) |
| **40–49** | 128 | 26 (20.3) |
| **50–59** | 103 | 49 (47.6) |
| **≥60** | 87 | 56 (64.4) |

**Table S2. Prevalence of hypertension by BMI category (N = 525).**

| BMI | Total, n | Hypertension, n (%) |
|---|---|---|
| **Underweight** | 9 | 1 (11.1) |
| **Normal** | 119 | 21 (17.6) |
| **Overweight** | 189 | 49 (25.9) |
| **Obese** | 208 | 75 (36.1) |

*BMI categories were defined as underweight (BMI <18.5 kg/m²), normal weight (18.5–24.9 kg/m²), overweight (25.0–29.9 kg/m²), and obesity (≥30.0 kg/m²).*
